# Near-Complete Genome Sequences of Eight Human Astroviruses Recovered from Diarrheal Stool Samples of Hospitalized Children in Coastal Kenya in 2019

**DOI:** 10.1128/MRA.00162-21

**Published:** 2021-04-15

**Authors:** Arnold W. Lambisia, My V. T. Phan, Zaydah R. de Laurent, Matthew Cotten, D. James Nokes, Charles N. Agoti

**Affiliations:** a Kenya Medical Research Institute (KEMRI)-Wellcome Trust Research Programme, Centre for Geographic Medicine Research-Coast, Kilifi, Kenya; b UK Medical Research Council, Uganda Virus Research Institute and London School of Hygiene and Tropical Medicine Uganda Research Unit, Entebbe, Uganda; c School of Life Sciences and Zeeman Institute (SBIDER), University of Warwick, Coventry, United Kingdom; d School of Health and Human Sciences, Pwani University, Kilifi, Kenya; KU Leuven

## Abstract

Here, using a sequence-independent sequencing approach (M. V. Phan, P. Hong Anh, N. Van Cuong, B. Oude Munnink, et al., Virus Evol 2:vew027, 2016, https://doi.org/10.1093/ve/vew027), we determined human astrovirus (HAstV) genome sequences from eight diarrheal stool samples collected in coastal Kenya in 2019. Phylogenetic analysis identified the following 4 genotypes: HAstV-1 (*n* = 4), HAstV-2 (*n* = 1), HAstV-3 (*n* = 1), and HAstV-5 (*n* = 2).

## ANNOUNCEMENT

Human astroviruses (HAstVs) (family *Astroviridae*) are nonenveloped, 7-kb positive-sense, single-stranded RNA genome viruses (1) and are among the top 5 viral causes of childhood diarrhea globally (2). HAstV clinical isolates are classified into classic HAstVs (HAstV-1 to HAstV-8), HAstV-MLB, and HAstV-VA/HMO (1).

In Kenya and other African settings, HAstV positivity in children with diarrhea as one of their illness symptoms ranges from 2.7% to 10.3% (3–5). To date, there are no complete or near-complete (≥90% genome coverage) HAstV genome sequences from East Africa in the GenBank database (6). Analysis of HAstV genome sequences may facilitate optimization of molecular diagnostics and tracking the spread of HAstVs (7). Here, we utilized sequence-independent single-primer amplification (SISPA) sequencing to generate new HAstV genome sequences from positive reverse transcription-quantitative PCR (RT-PCR) (5) samples collected from children hospitalized with diarrhea in Kilifi, Kenya.

Total nucleic acid (TNA) was extracted from the 10 stool specimens using the QIAamp fast DNA stool minikit (Qiagen, Manchester, United Kingdom). The TNA was treated with Turbo DNase (Invitrogen, Carlsbad, CA), and first-strand synthesis was performed with FR26RV-ENDOH primers (8). Second-strand DNA synthesis was performed with Klenow fragment 3′ to 5′ exo- (New England BioLabs). To achieve a nonselective nucleic acid amplification, double-stranded DNA (dsDNA) was primed with the FR20RV primer (5′-GCCGGAGCTCTGCAGATATC-3′), complementary to the FR26RV-ENDOH primers at the 5′ end (9), and amplified using SuperScript III with the Platinum *Taq* DNA polymerase kit (Qiagen) as per the manufacturer’s protocol. The PCR product was used to prepare Illumina barcoded libraries using the Illumina DNA Flex kit and sequenced in one run using the Illumina MiSeq machine generating 75-bp paired-end reads. Sequencing adapters and low-quality bases (Phred score,<30) were trimmed/removed from the short-read data using QUASR v.7.03 (10). Reference HAstV-1, HAstV-2, HAstV-3, and HAstV-5 genome sequences (GenBank accession numbers JF327666, KF039911, MN444721, and MF684776, respectively) were used for reference-guided assembly and to transfer annotations to the assembled genomes using the inbuilt Geneious mapper and annotation tools, respectively, on Geneious Prime v.2019.2.3 (11). MAFFT v.7.313 (12) was used for nucleotide coding sequence alignment, and maximum likelihood phylogenies were reconstituted in IQ-Tree v.2.0.6 (13) with standard model selection. Written informed consent for study participation was obtained from parents/guardians of the enrolled children, and the study protocol was approved by the KEMRI Scientific and Ethics Review Unit (SSC 2861 and SERU CGMRC/113/3624).

**FIG 1:**
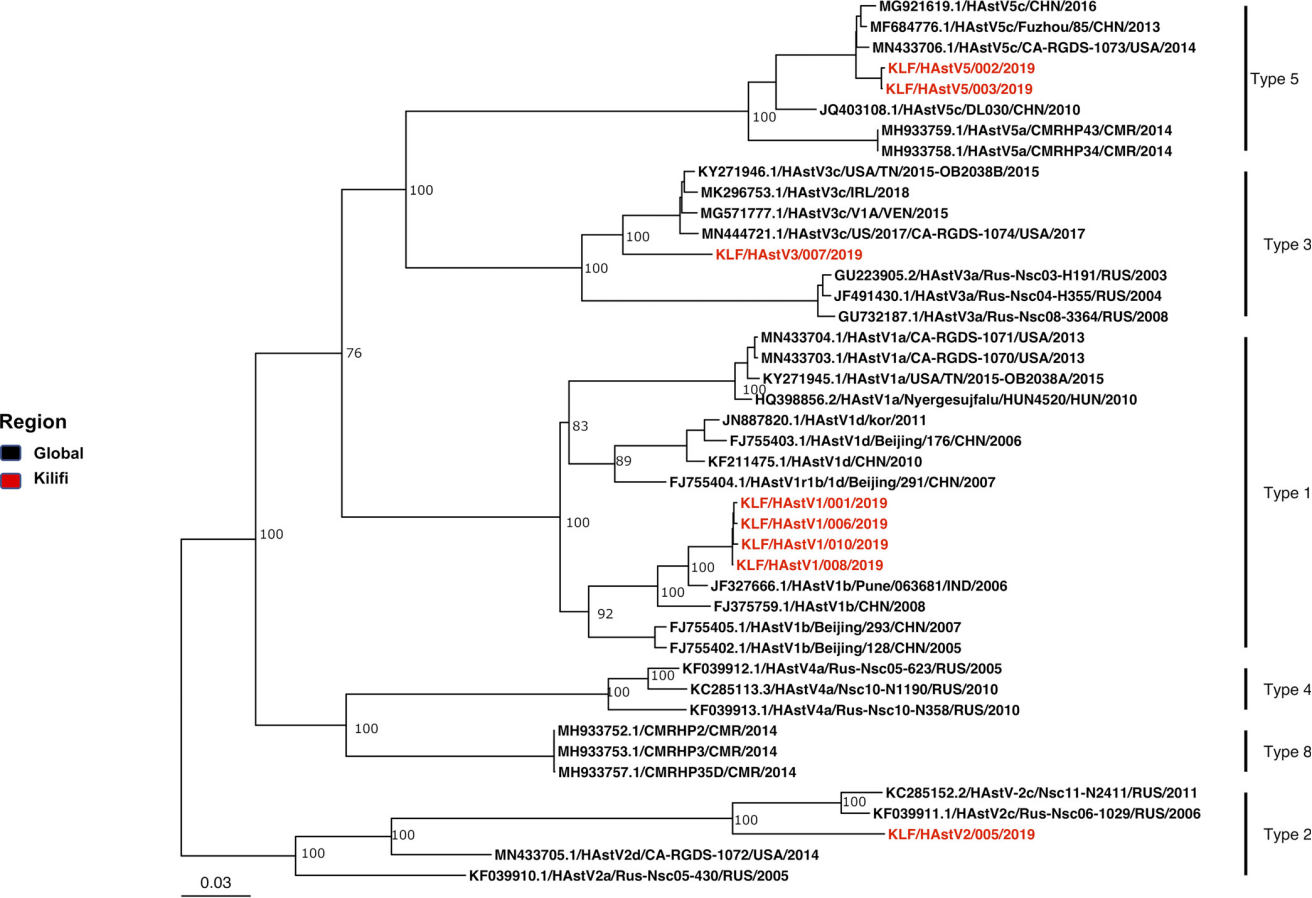
Maximum likelihood phylogenetic tree based on the open reading frame (ORF) sequences of the eight classical HAstVs (>90% genome coverage) identified in this study and representative strains from GenBank. The tree was constructed using IQ-Tree v.2.0.6 (13) with standard model selection. Bar indicates nucleotide substitutions per site. Red and black show HAstVs identified in this study and globally, respectively.

Patient demographics and sequencing output characteristics for the 10 samples are provided in Table 1. Eight samples yielded a consensus sequence covering >90% of the HAstV full-length genome. A maximum likelihood phylogeny of these eight near-complete genomes, including all publicly available HAstV genomes, is shown in Fig. 1. The new Kilifi sequences clustered with four different types of classical HAstVs, namely, HAstV-1 (*n* = 4), HAstV-2 (*n* =1), HAstV-3 (*n* = 1), and HAstV-5 (*n* = 2). Both the HAstV-1 (*n* = 4) and HAstV-5 (*n* = 2) genomes had > 99% nucleotide similarity within their respective types. These new near-complete HAstV genomes from coastal Kenya increase available HAstV genomic data to support future molecular studies and local diagnostic methods.

**TABLE 1:** Characteristics of human astrovirus genomes from coastal Kenya in 2019

Strain	Type	Collection date (day-mo-yr)	*C_T_* value^*a*^	Age (mo)	Sex	Symptom(s)^*b*^	Genome length (nt^*c*^)	Total no. of raw reads	No. of mapped reads	Avg depth^*d*^	Genome coverage (%)^*e*^	GC content (%)	Pairwise identity to reference (%)	GenBank accession no.	Reference genome length (nt)
KLF/ASV/001	HAstV1	13/4/2019	19.5	25	Female	D + V	6,115	2,014,832	179,076	138	90.24	44.3	97.2	MW485038	6,776
KLF/ASV/008	HAstV1	26/4/2019	22.8	23	Male	D + V	6,776	1,297,222	5,673	53	100.00	44.9	97.4	MW485040	6,776
KLF/ASV/010	HAstV1	18/7/2019	22.5	15	Male	D + V	6,398	1,317,294	878	9	94.42	47.9	97.2	MW485041	6,776
KLF/ASV/006	HAstV1	23/7/2019	21.4	8	Male	D + V	6,698	3,015,006	3,744	39	98.85	45.0	97.3	MW485039	6,776
KLF/ASV/009	HAstV1	10/6/2019	24.0	27	Female	D + V	5,342	1,912,848	388	5	78.84	6,776
KLF/ASV/004	HAstV1	19/6/2019	22.2	10	Female	D + V	4,788	1,952,244	195	3	70.66	6,776
KLF/ASV/005	HAstV2	19/6/2019	23.9	12	Male	D + V	6,725	1,581,106	13,121	158	99.22	44.2	90.3	MW485042	6,778
KLF/ASV/007	HAstV3	15/4/2019	26.2	22	Female	D	6,747	2,302,640	10,137	100	99.37	44.0	94.1	MW485043	6,790
KLF/ASV/002	HAstV5	1/6/2019	24.3	9	Female	D	6,666	2,906,060	1,769	18	97.99	43.6	98.3	MW485044	6,803
KLF/ASV/003	HAstV5	1/6/2019	22.4	24	Male	D + V	6,361	3,046,380	1,188	13	93.50	43.7	98.5	MW485045	6,803

a The real-time RT-PCR (rRT-PCR) assay, including primers and probe sequences used for HAstV detection, has been described previously (6). *C_T_*, cycle threshold.

b Objective evidence of a diarrheal disease. D, diarrhea; V, vomiting.

C nt, nucleotide.

d Calculated by dividing the per-position coverage output by respective genome length.

e Calculated by dividing the genome length by the respective reference genome length.

### Data availability.

The raw sequence data were deposited in the Sequence Read Archive (SRA) under BioProject accession number PRJNA692787 and BioSample accession numbers SAMN17370496 to SAMN17370503. The genome sequences generated here were deposited in GenBank under accession numbers MW485038 to MW485045.
